# J. N. Johnson

**Published:** 1823-11

**Authors:** 


Lately deceased, J. N. JOHNSON, M.D. Fellow of the Royal College of Physicians, &c.

